# Healthcare professionals’ views on implementing digital health tools in psychosis: a national survey in the UK

**DOI:** 10.1136/bmjdhai-2025-000005

**Published:** 2025-06-11

**Authors:** Xiaolong Zhang, Emily Eisner, Daniela Di Basilio, Cara Richardson, Joseph Firth, Sandra Bucci

**Affiliations:** 1 Division of Psychology and Mental Health, Faculty of Biology, Medicine and Health, Manchester Academic Health Science Centre, School of Health Sciences, The University of Manchester, Manchester, UK; 2 Greater Manchester Mental Health NHS Foundation Trust, Manchester, UK

**Keywords:** Health Services Research, Smartphone, Telemedicine

## Abstract

**Objective:**

Psychosis is a severe mental health problem and a huge public health challenge, but treatment is often delayed and inefficient. Digital health tools (DHTs) can support healthcare delivery and facilitate self-management. This study aimed to thoroughly understand staff members’ views and opinions on implementing DHTs to ensure such tools target unmet needs, are acceptable and maintain patient, service and public trust.

**Methods and analysis:**

A bespoke survey was developed to capture key issues surrounding implementation and acceptability of DHTs in SMI and rolled out nationally online to mental health staff working across 31 National Health Service sites in the UK.

**Results:**

We received 352 completed surveys. The results showed that most staff (74.2%) would like to implement DHTs in clinical practice. Regarding digital remote monitoring, staff felt less comfortable about passive sensing versus active symptom monitoring and felt less comfortable about tracking service users’ behaviours or whereabouts than tracking feelings or general health.

**Discussion:**

To our knowledge, this is the first national survey investigating staff views on digital mental health in psychosis in the UK. This study revealed new understanding of staff views on using DHTs in psychosis in general and using smartphone apps and wearables for remote monitoring and passive sensing specifically. Future studies are needed to optimise implementation strategies to address the barriers for technologies that have demonstrated feasibility, acceptability, usability, safety and effectiveness.

**What is already known on this topic**

- Service user acceptance and engagement with digital health systems is poor, which has, in part, been attributed to a lack of coproduction of these systems with all stakeholders. Mental health staff is one group of important stakeholders whose actions can ultimately determine whether a new technology is successfully adopted.

**What this study adds**

- This national survey revealed a new understanding of staff views on implementing digital mental health tools in psychosis in general and using smartphone apps and wearables for remote monitoring and passive sensing specifically. We found that most staff (74.2%) would like to implement digital health tools (DHT) in clinical practice. Staff felt less comfortable about passive sensing than active symptom monitoring, and more staff felt comfortable tracking feelings/general health than behaviours/whereabouts.

**How this study might affect research, practice or policy**

- While most staff supported the use of DHTs in clinical practice and felt that such technologies would benefit most service users on their case loads, concerns related to the digital divide, privacy and ethical issues, and risks of exacerbating symptoms still exist, suggesting future studies are needed to optimise implementation strategies to address the barriers.

## Introduction

Psychosis is a severe mental health problem and a huge public health challenge, with 80% of people experiencing at least one relapse within 5 years of a first episode.^1^ Each relapse is costly to health services^2^ and increases the individual’s risk of developing persistent psychotic symptoms and disconnection from education, employment and social development, adversely affecting long-term outcomes.^3^ However, local service structures are under immense pressure to keep up with demand. Long waiting times, the stigma of accessing mental health services and reactive care result in delayed and inefficient treatment. Additionally, conventional methods of monitoring and treating psychosis rely on patients recalling symptoms over the preceding week(s) or month(s) at a scheduled appointment time.^4 5^ Appointments are typically clinic based and often infrequent in overstretched mental health services, resulting in imprecise retrospective recall of symptoms.^6 7^ This is problematic in a condition that requires precise, time-sensitive treatment, particularly in response to signs of emerging relapse.

Digital health tools (DHT) can support healthcare delivery and facilitate self-management by allowing remote, low-cost mental health monitoring, with personalised intervention strategies delivered when needed. Advancements in digital technology, such as smartphones and wearables, can help improve the precision and promptness of symptom monitoring given their ability to generate a plethora of data through active and passive monitoring methods.^8^ Active symptom monitoring is typically defined as the user self-reporting data (eg, psychological, behavioural, physiological) by ecological momentary assessment or other ambulatory assessment methods.^9^ Passive sensing refers to using a smartphone and its embedded sensors or a wearable device to measure a user’s behaviour pattern (eg, app usage, activity log) or contextual information (eg, location) via passive collection of user data.^10^ By combining active symptom monitoring and passive sensing, smartphone-based monitoring has the potential to provide a more dynamic, personal and valid representation of a person’s emotional and behavioural state.^6 11^ Additionally, studies have indicated that active self-monitoring of symptoms can reduce positive psychotic symptoms in people with recent-onset psychosis.^12^

Implementation of new technologies in health services can be challenging. Service user acceptance and engagement with digital health systems is poor, which has, in part, been attributed to a lack of coproduction of these systems with all stakeholders.^13^ Greater stakeholder involvement at all levels of development and implementation is urgently needed to ensure that digital systems target unmet needs and maintain patient, service and public trust.^14^ Service users, mental health staff and service managers are important stakeholders whose actions can ultimately determine whether a new technology is successfully adopted. Previous survey studies have suggested that mental health staff working with people with bipolar disorder,^15^ young people^16^ and people with severe mental health problems in low- and middle-income countries^17^ have a relatively high rate of personal use of digital technologies, are interested in using DHTs in clinical practice and, for some, have already used DHTs to support clinical care. Additionally, qualitative investigations have been conducted to explore staff views on using DHTs with people with severe mental health problems in clinical practice.^18 19^ Despite this, there is a dearth of large-scale, quantitative research exploring staff members’ views and opinions on implementing DHTs in psychosis specifically.

Hence, the current study focused on gathering detailed survey data from mental health staff to explore their views about: (1) how DHTs can be implemented into mental health services; (2) strengths and challenges of deploying DHTs; and (3) utility and acceptability of complex digital remote monitoring systems using active and passive symptom monitoring methods. In this paper, we present the results of healthcare professionals’ views regarding implementing DHTs in clinical routine practice and using smartphone apps and wearable devices for active symptom monitoring and passive sensing in psychosis specifically.

## Materials and methods

### Participants

This was a one-off survey study with mental health staff working within the National Health Service (NHS) in the UK. We intentionally included staff from all professional backgrounds to capture a broad and diverse range of views. Eligibility criteria were: (1) working within an NHS service providing mental health support to people who experience psychosis or severe mental health problems; (2) 18 years or older; and (3) ability to provide informed consent. Participants not sufficiently fluent in English to complete the survey were excluded from participation. To reach a broad sample, we employed a wide range of recruitment methods, including online platforms (eg, social media platforms such as X and Reddit), presentations to clinical teams at team meetings and/or circulation of study information material via email or in person, and through the Clinical Research Network across England. All participants had the option to enter a £20 prize draw at the end of the survey as a thank you for their time.

### Ethics statement

The authors assert that all procedures contributing to this work comply with the ethical standards of the relevant national and institutional committees on human experimentation and with the Helsinki Declaration of 1975, as revised in 2013. Written informed consent was provided by all participants.

### Patient and public involvement

Clinicians contributed by reviewing and refining the phrasing of survey items.

### Data collection

The survey was conducted from November 2022 to March 2024. Demographic information and staff views on using DHTs in clinical practice and barriers to implementing these tools were collected. The survey questions reported in this paper are shown in online supplemental table 1. The survey was developed based on a review of existing literature on DHTs in mental healthcare,^20 21^ as well as input from experts in digital mental health and clinical practice. Items were designed to capture key themes relevant to the implementation and acceptability of DHTs, including perceived usability, and the strengths and challenges of adoption. To assess broad patterns of clinician attitudes, survey items grouped related concepts rather than specifying individual data types; for example, instead of examining clinician comfort with specific data types (eg, mood vs step count vs heart rate), we assessed comfort with remote monitoring in clinical practice by distinguishing between *feelings or general health* and *behaviours or whereabouts*. This approach was applied throughout the survey to balance clarity with flexibility, enabling respondents to interpret questions within their clinical context while maintaining a focus on overarching themes in digital health adoption. Survey items were reviewed by two clinicians to assess clarity and relevance of items. Feedback from this review led to minor refinements, such as rephrasing certain items for clarity. No substantial changes were required following this review. The whole survey took approximately 20–30 min to complete. Participants had the option to complete an online or paper version of the questionnaire. The online survey was delivered using Qualtrics.^22^ A URL link or a QR code was used to access the online survey. The participant information sheet (PIS) with the study information was embedded in the survey. After reading the PIS, participants completed screening questions to assess their eligibility. If not eligible, participants were shown a thank you message explaining that they could not take part in the survey as they did not fully meet the criteria for participation, and the survey ended; if eligible, participants were taken to the survey questions. For participants using a paper questionnaire, a study pack with a paper survey and return postage-paid envelope was sent. The content of the paper version was identical to the online version. The answers from the paper questionnaires were entered manually into the Qualtrics database.

10.1136/bmjdhai-2025-000005.supp1 Supplementary data

### Data analysis

Descriptive statistics were carried out using the R program^23^ to analyse quantitative data. Data visualisation was created using the R program and Microsoft Excel.^24^ Free-text answers were summarised narratively. When a large volume of data was obtained in any of these fields, we categorised and counted similar content using NVivo V.12.^25^ The proportion of missing rates for each question was summarised and reported in the results.

## Results

### Participant characteristics

We received 537 responses; of these, 352 completed the survey, which resulted in a completion rate of 65.55%. Demographic characteristics of the final sample are shown in table 1. The median age of the sample was 40 years (IQR 31–50), and the median time working in current role was 4 years (IQR 1.5–10). Most of the sample were female (n=244/352, 69.32%), white ethnicity staff (n=276/352, 78.41%) and with a university bachelor’s degree (n=129/352, 36.65%). Around a third were practising as mental health nurses (n=99/352, 28.12%).

**Table 1:** Characteristics of survey respondents (n=352)

Characteristics	Values
Age, median (IQR**)**	40 (31–50)
Gender, n (%)	
Female	244 (69.32)
Male	102 (28.98)
Non-binary/third gender	1 (0.28)
Prefer not to say/unsure	5 (1.42)
Ethnicity, n (%)	
Indian	19 (5.40)
Pakistani	11 (3.12)
Bangladeshi	4 (1.14)
Chinese	3 (0.85)
Any other Asian background	8 (2.27)
Caribbean	1 (0.28)
African	17 (4.83)
Any other black, black British or Caribbean background	2 (0.57)
White and black Caribbean	2 (0.57)
English, Welsh, Scottish, Northern Irish or British	244 (69.32)
Irish	6 (1.70)
Gypsy or Irish Traveller	1 (0.28)
Any other white background	25 (7.10)
Arab	1 (0.28)
Any other ethnic group	3 (0.85)
Any other mixed or multiple ethnic background	3 (0.85)
Prefer not to say	2 (0.57)
Highest education received, n (%)	
Secondary school	7 (1.99)
Diploma or equivalent	41 (11.65)
Trade/technical/vocational training	6 (1.70)
University bachelor’s degree	129 (36.65)
University master’s degree	118 (33.52)
PhD or higher	47 (13.35)
Prefer not to say	1 (0.28)
Missing	3 (0.85)
Job title, n (%)	
Mental health nurse	99 (28.12)
Social worker	13 (3.69)
Support worker/nursing assistant/STR worker/healthcare assistant	37 (10.51)
Psychotherapist (or trainee)	11 (3.12)
Psychiatrist (consultant, trainee, non-training grade)	50 (14.20)
Psychologist (qualified, assistant, trainee)	54 (15.34)
Allied health professional	28 (7.95)
Student nurse/student social worker/medical student/student allied health professional	7 (1.99)
Prefer not to say	9 (2.56)
Other	43 (12.22)
Missing	1 (0.28)
Years working in current role, median (IQR**)**	4 (1.5–10)

### Implementing DHTs into clinical practice

When asked if participants would like to see DHTs implemented or used in their service, nearly three-quarters of participants reported ‘yes’ (74.2%, n=261/352), and only a small proportion reported ‘no’ (2.6%, n=9/352) or ‘unsure’ (21.3%, n=75/352). For those who reported ‘yes’, smartphone apps and wearables were the top tools they would like to see implemented (see online supplemental table 2 for a full list of the tools identified). Participants indicated the reasons behind their choices in free-text questions. Main reasons for supporting using DHTs were ‘it could benefit service users’, ‘it could support service provision and cope with staff shortage’, ‘it could provide more information and objective data’ and ‘it could promote self-management’, whereas most mentioned reasons for not using DHTs or being unsure about it were ‘need more information and evidence’, ‘not cost-effective’ and ‘not useful’. A full list of the reasons is shown in online supplemental tables 3 and 4.

**Figure 1:**
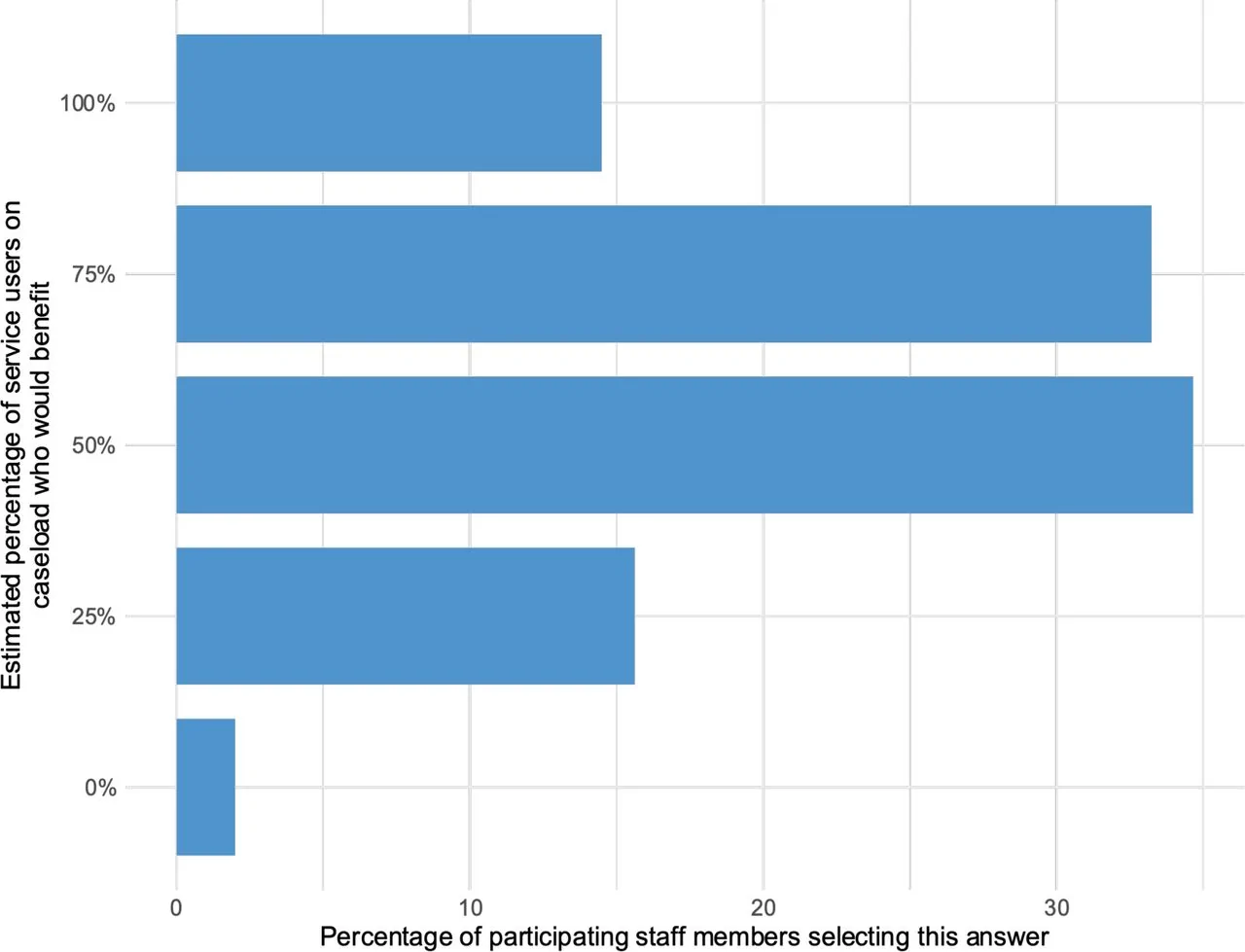
Participating staff members’ estimates of the percentage of people on the case load who would benefit from digital health tools to support mental health.

As shown in figure 1, two-thirds (n=239/352, 67.9%) of participants believed that 50–75% of the service users on their case load could benefit from using DHTs to support their mental health. Of note, 14.5% (n=51/352) indicated their whole case load could benefit from such tools. For those who reported that not all of their case load could benefit (ie, selected 75% or less), the main reasons were ‘service users not able to use DHTs’, ‘paranoia associated with technology’, ‘poor engagement’ and ‘may be distressed by using technology’ (see online supplemental table 5 for the full list).

**Figure 2:**
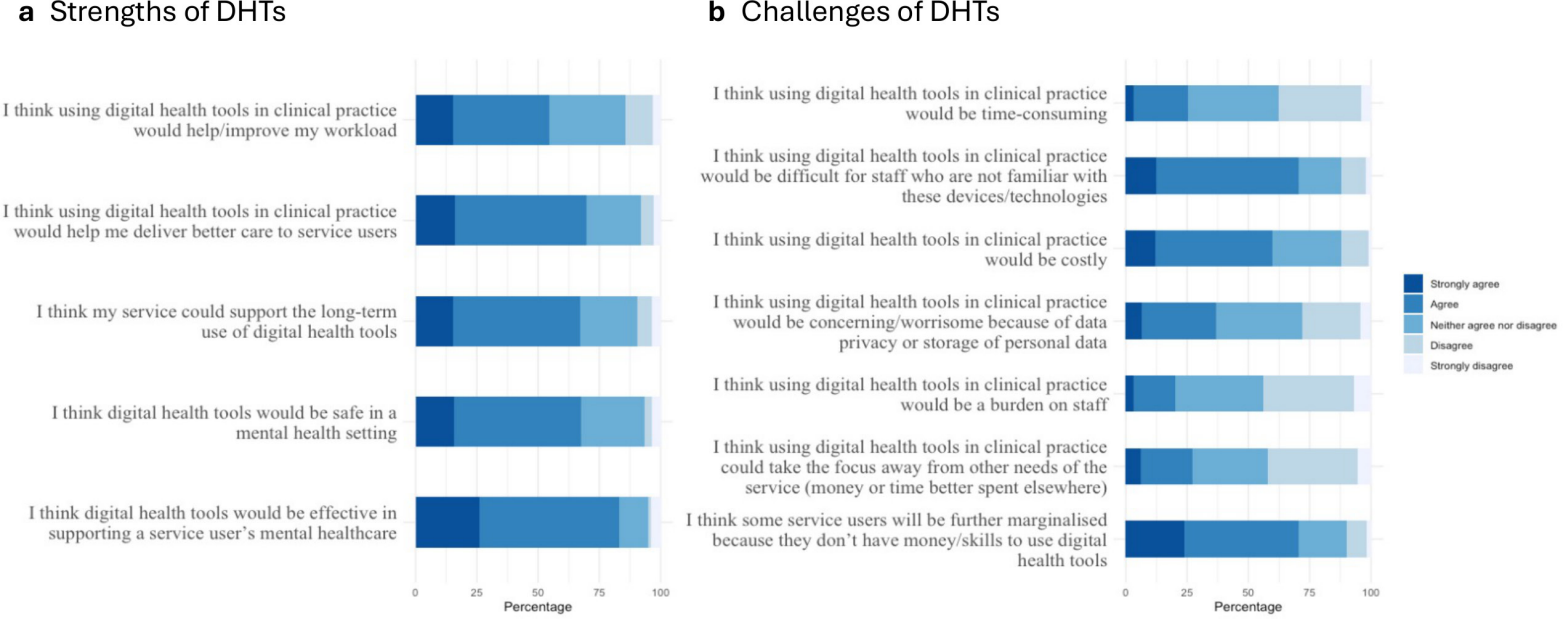
Staff members’ opinions on the strengths (A) and challenges (B) of digital health tools (DHTs).

Figure 2 shows staff members’ opinions on the strengths and challenges of DHTs in general. Regarding the strengths of using DHTs in clinic, 82.95% (n=292/352) strongly agreed or agreed that DHTs would be effective in supporting a service user’s mental healthcare. More than three-quarters of staff either strongly agreed or agreed that DHTs would be safe (n=238/352, 67.61%), could be used in the long term (n=236/352, 67.05%) and would help deliver better care (n=245/352, 69.6%). In terms of challenges, around 70% of respondents worried that DHTs might further marginalise people who do not own/use digital technologies (n=248/352) and that some staff might find it difficult to use DHTs (n=248/352), and around 60% (n=211/352) felt using DHTs in practice would be costly.

**Figure 3:**
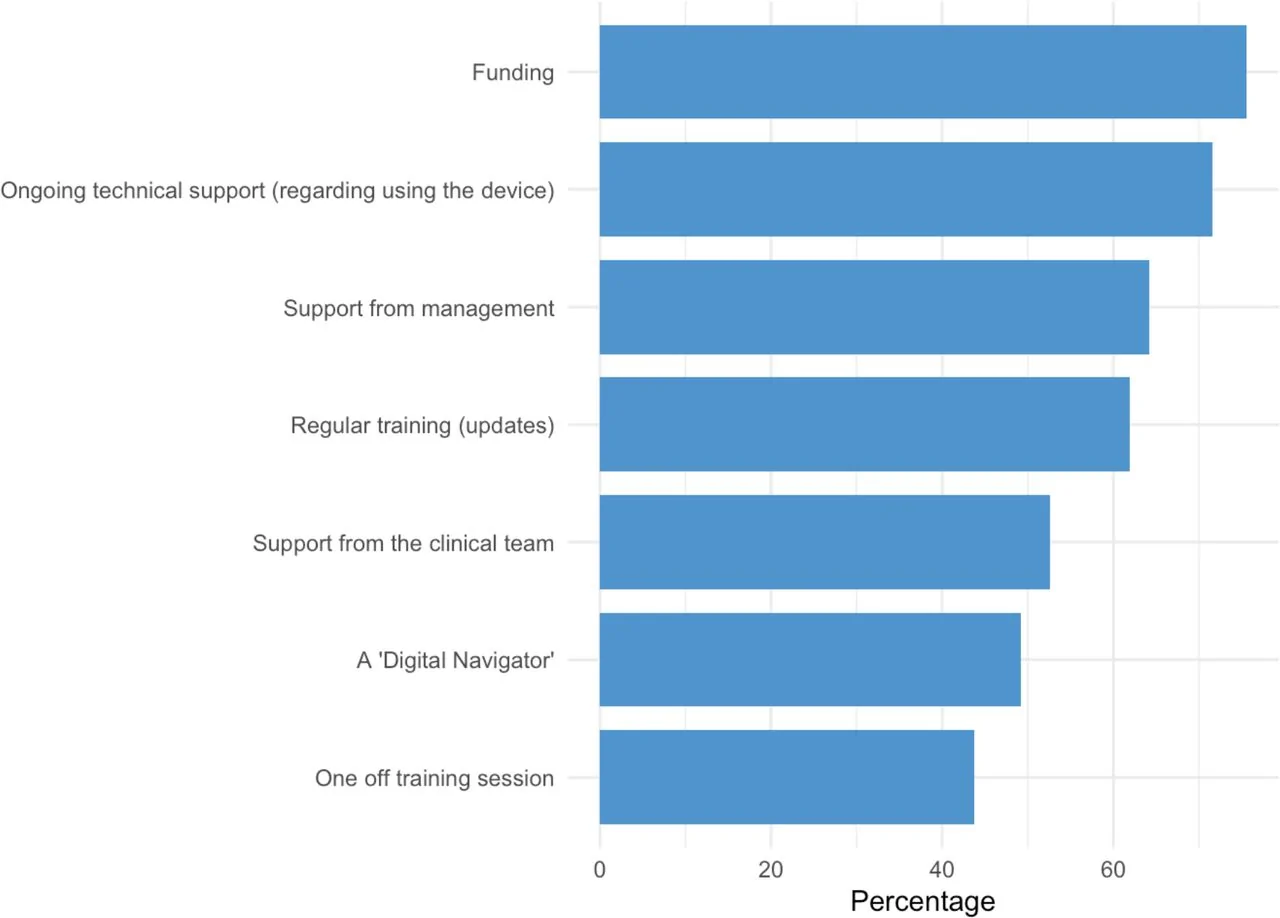
Training or infrastructure needed to support the use of digital health tools in clinical practice.

Regarding training or infrastructure required for using DHTs in clinical practice (see figure 3), most needed supports were funding (75.6%, n=266/352), ongoing technical support (71.6%, n=252/352), support from management (64.2%, n=226/352) and regular training (61.9%, n=218/352). One additional training need, extracted from free-text data, was ‘a library of digital clinical resources or a website for information and support’.

### Views on smartphone apps and wearables

**Figure 4:**
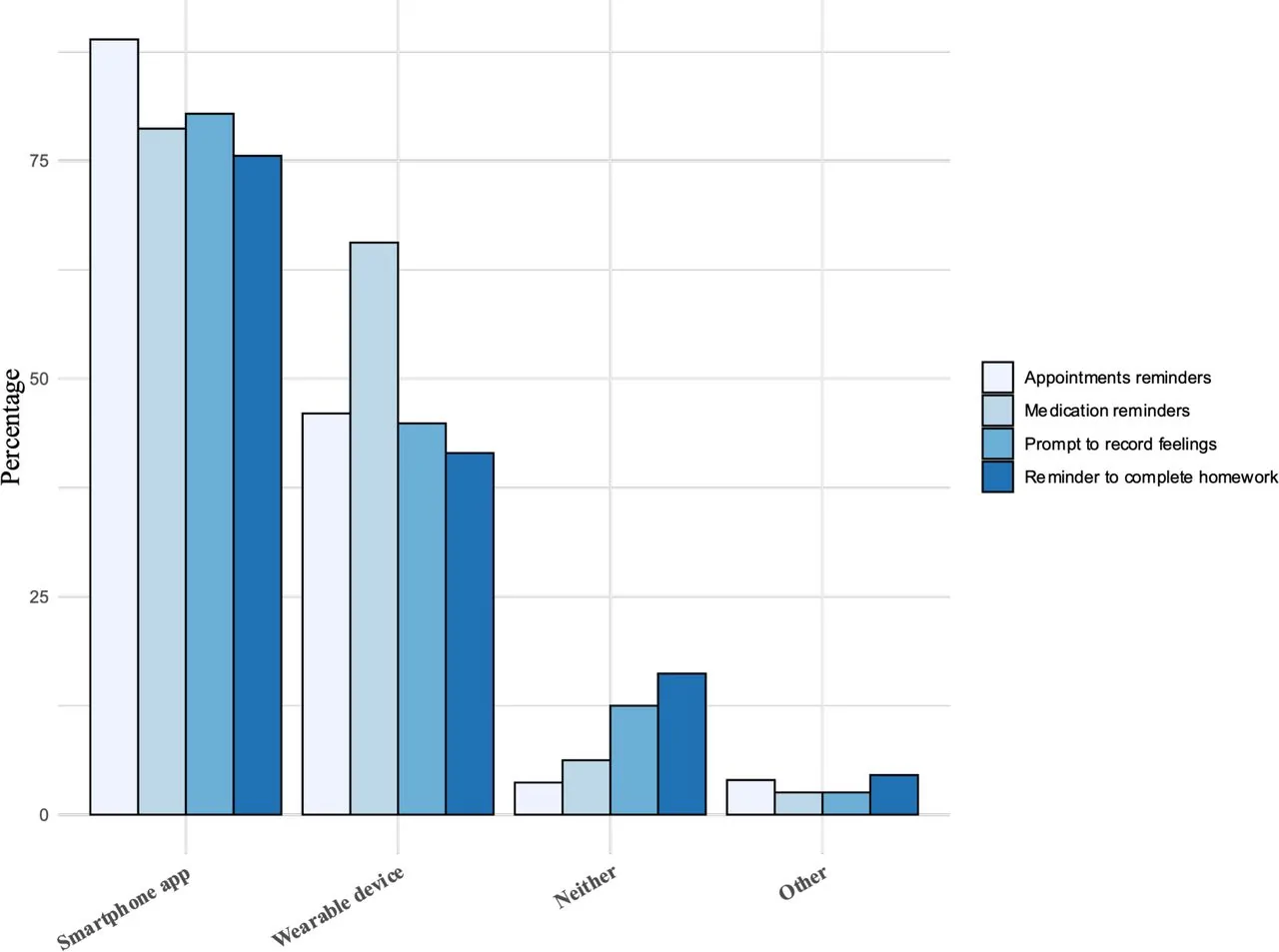
Functions that participants would like to see digital health tools used for in clinical practice.

Figure 4 shows the functions participants would like to see DHTs used for in clinical practice. Most participants endorsed using smartphone apps to help service users to remember healthcare appointments (n=313/352, 88.92%) and wearable devices to alert or remind service users to take medication (n=231/352, 65.63%). In general, more participants endorsed using smartphone apps than wearable devices for all proposed functions. We do, however, acknowledge that the use cases provided for this item were limited and not exhaustive, which may have influenced participants’ responses. A broader selection of potential applications might have yielded different preferences, reflecting a wider range of clinician priorities.

As shown in online supplemental table 6, the pattern of participants’ views on the potential advantages of apps and wearables was similar; however, fewer people endorsed ‘agree’ or ‘strongly agree’ and more felt ‘neutral’ about the advantages of wearables compared with smartphone apps. A significant majority of participants reported ‘agree’ or ‘strongly agree’ that smartphone apps provide service users with the opportunity to record and reflect on symptoms and experiences over time (86.37%, n=304/352), identify triggers and patterns (86.08%, n=303/352) and share information in ‘real-time’ with their clinical team (78.13%, n=275/352), and that such digital tools can help service users take control of their mental health (73.01%, n=257/352). In contrast, fewer people expressed ‘agree’ or ‘strongly agree’ for wearable devices on these potential advantages, with the percentage being 66.48% (n=234/352), 70.17% (n=247/352), 63.92% (n=225/352) and 56.54% (n=199/352), respectively. A considerable proportion of participants felt ‘neutral’ that service users may feel that information in smartphone apps (36.65%, n=129/352) and wearable devices (36.93%, n=130/352) is more private compared with talking to a member of their clinical team.

### Opinions on the use of active symptom monitoring and passive sensing in clinical practice

Online supplemental table 7 shows respondents’ opinions on using active symptom monitoring and passive sensing in clinical practice. Most respondents felt neutral about potential implementation issues (eg, implementing it would be too costly; see online supplemental table 7 for details); however, most participants either agreed or strongly agreed that active symptom monitoring would be difficult to use for staff (n=214/352, 60.79%) or service users (n=285/352, 73.3%) who are not familiar with DHTs. For passive sensing, the percentage was 62.22% (n=219/352) for staff and 60.23% (n=212/352) for service users.

In terms of tracking feelings, general health, behaviours or whereabouts via active symptom monitoring, most staff were comfortable with tracking feelings or general health (n=208/352, 58.24%), whereas fewer staff felt comfortable (n=146/352, 41.48%) about tracking behaviours or whereabouts. Compared with this, fewer staff were comfortable with tracking feelings or general health (n=146/352, 41.48%) via passive sensing, and even fewer staff felt comfortable (n=119/352, 33.81%) about using it for tracking behaviours or whereabouts.

We asked staff to report their concerns and comments regarding using remote symptom monitoring and passive sensing in clinical practice in a free-text question. Top concerns about active symptom monitoring were ‘capacity to respond’, ‘monitoring feels intrusive and controlling’ and ‘it may increase service users’ paranoia or anxiety about tech’. For passive sensing, participants emphasised consent issues, with concerns expressed that service users may not be able to fully understand the concept of passive sensing. Similar to active symptom monitoring, staff felt passive sensing was intrusive and controlling and could increase paranoia and anxiety. The full list of concerns and comments on remote symptom monitoring and passive sensing is displayed in online supplemental tables 8 and 9.

## Discussion

Given the survey was conducted shortly after the COVID-19 pandemic, a period that significantly accelerated the adoption and development of DHTs, this study provides timely information on the views of mental health professionals in the UK regarding implementing DHTs in routine clinical practice and using smartphone apps and wearable devices for active symptom monitoring and passive sensing in psychosis specifically. We used both online and paper versions of the survey to reach a broader sample. We found that a significant majority (74.2%) of staff would like to implement DHTs in clinical practice. Most staff (67.9%) deemed 50–75% of their case load could benefit from DHTs and felt DHTs would be effective and safe and would improve their workload. In terms of active symptom monitoring and passive sensing, staff felt less comfortable about passive sensing versus active symptom monitoring and felt less comfortable about tracking service users’ behaviours or whereabouts than tracking feelings or general health. To our knowledge, this is the first national survey investigating staff views on digital mental health in psychosis in the UK.

Despite the overall positive views, staff also expressed concerns related to service users’ ability to use DHTs, paranoia associated with technology, poor engagement and risk of distress by using technology, which may result in service users not benefiting from DHTs. Nonetheless, a recent review study supported the safety of DHTs in psychosis,^26^ suggesting many of these concerns among staff could be addressed with education and recommendations as to how DHTs can be used safely in this group.

Another concern raised was how approximately 70% of staff felt DHTs might widen the digital divide. That said, a recent UK-based survey study showed that digital device ownership in people with psychosis was high and increased rapidly in the past years, with the most recent data being 90% owning smartphones and 95% owning mobile phones.^27^ In addition, given the affordability of smartphones, it is feasible to provide devices to people who would benefit from DHTs but who cannot afford one. For example, NHS City and Hackney has trialled using the personal health budget to purchase digital devices for people with severe mental health problems to access the digital recovery platform.^28^ Therefore, some staff concerns reported in the survey reflected a knowledge gap about DHTs in psychosis.

Training is considered one way to improve staff digital health literacy and, in turn, increase staff acceptance of DHTs.^29–31^ In the UK, NHS England has established the NHS Digital Academy to deliver training programmes such as the Digital Readiness Education programme to build digital skills, knowledge, understanding and awareness across the health and care workforce.^32^ NHSX (now part of the NHS Transformation Directorate) launched the mental health digital playbook in 2021, aimed to support clinical teams and organisations for digitally enabled mental health pathways.^33^ However, only a small proportion of the content of these NHS training programmes is related to severe mental health problems. This may explain the knowledge gap identified by our survey. A knowledge gap in digital skills among mental health professionals has also been identified in other countries, such as the USA^34^ and Europe.^35^ While many of the identified gaps are common across DHT, our focus on psychosis highlights the need for tailored training that accounts for the unique complexities of this condition; however, future research comparing these gaps across specialties would help determine whether the gaps are truly distinct or part of a broader pattern.

Funding was the most frequently mentioned factor required for using DHTs in clinical practice by participants. Lack of funding has led to information technology infrastructure in many NHS settings being outdated and not able to support the implementation of DHTs.^31^ Additionally, funding for research projects is fragmented and falls short of consideration of the complexity of science, often only covering the initial phases of DHT development, which has resulted in a lack of resources to support the rest of the implementation pathway.^36 37^ A recent review found that nearly half of apps developed for schizophrenia research in the last decade cannot be accessed online following the completion of their respective trials.^38^ Furthermore, given the nature of digital technologies, ongoing investment for maintaining and updating DHTs and regular cybersecurity and data protection measures is another significant cost.^36^ To facilitate the implementation of DHTs in clinical practice, sustainable investment in updating digital infrastructure and long-term research is needed.^31 36^

Smartphone apps and wearable devices are the main technologies used to enable digital remote monitoring.^8^ The current survey found that most healthcare staff considered smartphone apps and wearable devices to be helpful for helping service users towards recording and reflecting on symptoms and experiences (apps 86.37%, wearables 66.48%), identifying triggers and patterns (apps 86.08%, wearables 70.17%), sharing information in ‘real-time’ with the care team (apps 78.13%, wearables 63.92%) and helping take control of their mental health, including gaining greater autonomy in symptom monitoring, accessing self-management tools or feeling more empowered in decision-making about their care (apps 73.01%, wearables 56.54%). Notably, the respondents also felt that using smartphone apps to manage appointments and wearables for medication reminders would be more useful than recording feelings. This might reflect that staff were less aware of evidence about the feasibility and effectiveness of using apps and wearables for symptom monitoring compared with more straightforward applications such as using it as a reminder or alarm for managing healthcare appointments or taking medication. Of note, although participants’ views on the potential advantages of using apps and wearables in mental health settings were analogous, fewer participants endorsed ‘agree’ or ’strongly agree’ and more felt ‘neutral’ about the advantages of wearables compared with smartphone apps. This might relate to most wearable devices being advertised as a tool for monitoring physical activity^39^; staff may associate the tool more with physical health management rather than mental health management.^40^

Active symptom monitoring and passive sensing have the potential to increase the precision of symptom monitoring and support digital interventions to be delivered in a ‘right intervention at the right time’ manner.^8^ However, a noticeable proportion of people felt using such technologies could be time consuming (38.64%), burdensome (32.39%) or costly (31.25%), and considered money or time better spent elsewhere (26.99%). Moreover, in free-text responses, some staff expressed concerns that DHTs may be used to replace face-to-face care. This highlights that continuous effort is needed to ensure staff recognise the actual benefits of active symptom monitoring and passive sensing rather than viewing them as an additional burden or feeling threatened. Privacy is another major concern, especially around recording locations and phone logs of users. This is in line with previous studies, which also found that staff expressed concerns that intensive monitoring is intrusive and could potentially damage therapeutic relationships.^19 29^ One way to address this concern is to clearly explain the purpose of collecting data protection measures to users, allowing them to decide what data to share, when to share it and with whom.^41^ While making such data-sharing decisions may be burdensome for some individuals, particularly those who are acutely unwell, this approach prioritises informed consent, ensuring that those with the capacity to make such choices are given these options. More importantly, a clear guideline for good practice of digital remote monitoring (eg, data protection protocols and transparency) is needed to address the ethical and legal concerns.

One strength of this study is that we recruited a national sample across 31 NHS sites in the UK. Additionally, we included specific questions about using smartphone apps and wearables and active symptom monitoring and passive sensing, which afforded a comprehensive understanding of staff views on digital remote monitoring, a rapidly growing area of digital mental health. However, this study has some limitations. First, although we recruited a national sample, it was a convenience sample; therefore, we cannot guarantee the representativeness of participants. Second, although we used both paper and online versions to ensure people both familiar and not familiar with technologies were included, given the nature of a self-selected sample, some participants may have been more familiar, interested and comfortable with using digital tools than is typical. Moreover, given some participants had little knowledge and experience of DHTs, this survey largely captured the ‘hypothetical’ views of these participants, which might change after staff have more experience of using DHTs in clinical practice. Furthermore, one potential consideration is the emergence of generative artificial intelligence technologies during the survey data collection period. However, as our study primarily focused on smartphones, wearable devices and remote symptom monitoring, areas less directly impacted by these advancements, we do not anticipate this technology had a substantial influence on our findings. Also, while staff endorsed the item that DHTs are safe for use in mental healthcare, it is uncertain whether all participants interpreted ‘safety’ uniformly. While we did not define ‘safety’ explicitly in the survey, the broad question allowed participants to highlight the aspects they found most relevant. Given their clinical expertise, participants likely considered risks such as symptom exacerbation, distress, reduced engagement with care, data privacy concerns, ethical issues and their familiarity with regulations and broader discussions on DHTs risks and benefits when responding to the safety item. Finally, although beyond the scope of this study, future studies should compare the views of staff and service users on DHTs to identify potential discrepancies and assess whether these differences might hinder the implementation of DHTs in clinical practice.

The adoption curve is a common phenomenon for healthcare innovations,^42^ and implementing DHTs in psychosis is no exception. This study revealed a new understanding of staff views on using DHTs in psychosis in general and using smartphone apps and wearables for remote monitoring and passive sensing specifically. The results indicated a generally positive attitude among staff, with the majority supporting the use of DHTs in clinical practice and believing these technologies would benefit most service users on their case loads. However, despite evidence in the wider literature on the feasibility and safety of DHTs in psychosis, concerns related to the digital divide, privacy and ethical issues, and risks of exacerbating symptoms still exist, suggesting continuous updating of evidence is needed to refresh staff knowledge about DHTs. Moreover, future studies are needed to optimise implementation models that could support the integration of DHTs into clinical workflow.

## Data Availability

Data are available upon reasonable request. The datasets used and/or analysed during the current study are available from the corresponding author on reasonable request.

## References

[1] Alvarez-Jimenez M , Priede A , Hetrick SE , et al . Risk factors for relapse following treatment for first episode psychosis: A systematic review and meta-analysis of longitudinal studies. Schizophr Res 2012;139:116–28. 10.1016/j.schres.2012.05.007 22658527

[2] Ascher-Svanum H , Zhu B , Faries DE , et al . The cost of relapse and the predictors of relapse in the treatment of schizophrenia. BMC Psychiatry 2010;10:2. 10.1186/1471-244X-10-2 20059765 PMC2817695

[3] Penn DL , Waldheter EJ , Perkins DO , et al . Psychosocial treatment for first-episode psychosis: a research update. Am J Psychiatry 2005;162:2220–32. 10.1176/appi.ajp.162.12.2220 16330584

[4] Andrade LH , Alonso J , Mneimneh Z , et al . Barriers to mental health treatment: results from the WHO World Mental Health surveys. Psychol Med 2014;44:1303–17. 10.1017/S0033291713001943 23931656 PMC4100460

[5] RCPsych . Two-fifths of patients waiting for mental health treatment forced to resort to emergency or crisis services. RCPsych; 2020. Available: https://www.rcpsych.ac.uk/news-and-features/latest-news/detail/2020/10/06/two-fifths-of-patients-waiting-for-mental-health-treatment-forced-to-resort-to-emergency-or-crisis-services [accessed 21 Oct 2021]

[6] Ben-Zeev D , Brian R , Wang R , et al . CrossCheck: Integrating self-report, behavioral sensing, and smartphone use to identify digital indicators of psychotic relapse. Psychiatr Rehabil J 2017;40:266–75. 10.1037/prj0000243 28368138 PMC5593755

[7] Insel TR . Digital phenotyping: a global tool for psychiatry. World Psychiatry 2018;17:276–7. 10.1002/wps.20550 30192103 PMC6127813

[8] Torous J , Bucci S , Bell IH , et al . The growing field of digital psychiatry: current evidence and the future of apps, social media, chatbots, and virtual reality. World Psychiatry 2021;20:318–35. 10.1002/wps.20883 34505369 PMC8429349

[9] Trull TJ , Ebner-Priemer U . Ambulatory assessment. Annu Rev Clin Psychol 2013;9:151–76. 10.1146/annurev-clinpsy-050212-185510 23157450 PMC4249763

[10] Trifan A , Oliveira M , Oliveira JL . Passive Sensing of Health Outcomes Through Smartphones: Systematic Review of Current Solutions and Possible Limitations. JMIR Mhealth Uhealth 2019;7:e12649. 10.2196/12649 31444874 PMC6729117

[11] Torous J , Wisniewski H , Bird B , et al . Creating a Digital Health Smartphone App and Digital Phenotyping Platform for Mental Health and Diverse Healthcare Needs: an Interdisciplinary and Collaborative Approach. J technol behav sci 2019;4:73–85. 10.1007/s41347-019-00095-w

[12] Lewis S , Ainsworth J , Sanders C , et al . Smartphone-Enhanced Symptom Management In Psychosis: Open, Randomized Controlled Trial. J Med Internet Res 2020;22:e17019. 10.2196/17019 32788150 PMC7453320

[13] Bucci S , Schwannauer M , Berry N . The digital revolution and its impact on mental health care. Psychol Psychother 2019;92:277–97. 10.1111/papt.12222 30924316

[14] Berry N , Machin M , Ainsworth J , et al . Developing a Theory-Informed Smartphone App for Early Psychosis: Learning Points From a Multidisciplinary Collaboration. Front Psychiatry 2020;11:602861. 10.3389/fpsyt.2020.602861 33362612 PMC7758439

[15] Morton E , Torous J , Murray G , et al . Using apps for bipolar disorder - An online survey of healthcare provider perspectives and practices. J Psychiatr Res 2021;137:22–8. 10.1016/j.jpsychires.2021.02.047 33647725

[16] Bell IH , Thompson A , Valentine L , et al . Ownership, Use of, and Interest in Digital Mental Health Technologies Among Clinicians and Young People Across a Spectrum of Clinical Care Needs: Cross-sectional Survey. JMIR Ment Health 2022;9:e30716. 10.2196/30716 35544295 PMC9133993

[17] Zhang X , Lewis S , Chen X , et al . Mental health professionals views and the impact of COVID-19 pandemic on implementing digital mental health in China: A nationwide survey study. Internet Interv 2022;30:100576. 10.1016/j.invent.2022.100576 36185346 PMC9509019

[18] Berry N , Bucci S , Lobban F . Use of the Internet and Mobile Phones for Self-Management of Severe Mental Health Problems: Qualitative Study of Staff Views. JMIR Ment Health 2017;4:e52. 10.2196/mental.8311 29092809 PMC5688247

[19] Bucci S , Berry N , Morris R , et al . “They Are Not Hard-to-Reach Clients. We Have Just Got Hard-to-Reach Services.” Staff Views of Digital Health Tools in Specialist Mental Health Services. Front Psychiatry 2019;10:344. 10.3389/fpsyt.2019.00344 31133906 PMC6524662

[20] Gipson S , Torous J , Boland R , et al . Mobile Phone Use in Psychiatry Residents in the United States: Multisite Cross-Sectional Survey Study. JMIR Mhealth Uhealth 2017;5:e160. 10.2196/mhealth.7146 29092807 PMC5688243

[21] Torous J , Franzan J , O’Connor R , et al . Psychiatry Residents’ Use of Educational Websites: A Pilot Survey Study. Acad Psychiatry 2015;39:630–3. 10.1007/s40596-015-0335-8 26077007

[22] Qualtrics . Qualtrics. 2024.

[23] R Core Team . R: a language and environment for statistical computing. 2021.

[24] Microsoft Corporation . Microsoft Excel. 2018.

[25] QSR International Pty Ltd . NVivo (version 12). 2018.

[26] Allan S , Ward T , Eisner E , et al . Adverse Events Reporting in Digital Interventions Evaluations for Psychosis: A Systematic Literature Search and Individual Level Content Analysis of Adverse Event Reports. Schizophr Bull 2024;50:1436–55. 10.1093/schbul/sbae031 38581410 PMC11548921

[27] Eisner E , Berry N , Bucci S . Digital tools to support mental health: a survey study in psychosis. BMC Psychiatry 2023;23:726. 10.1186/s12888-023-05114-y 37803367 PMC10559432

[28] NHS Transformation Directorate . A digital recovery platform for severe mental illness. NHS Transform. Dir; 2021. Available: https://transform.england.nhs.uk/key-tools-and-info/digital-playbooks/mental-health-digital-playbook/a-digital-recovery-platform-for-severe-mental-illness/ [Accessed 28 Jun 2024].

[29] Rogan J , Bucci S , Firth J . Health Care Professionals’ Views on the Use of Passive Sensing, AI, and Machine Learning in Mental Health Care: Systematic Review With Meta-Synthesis. JMIR Ment Health 2024;11:e49577. 10.2196/49577 38261403 PMC10848143

[30] Smith KA , Blease C , Faurholt-Jepsen M , et al . Digital mental health: challenges and next steps. BMJ Ment Health 2023;26:e300670. 10.1136/bmjment-2023-300670 PMC10231442 37197797

[31] techUK . The techuk ten point plan for healthtech. 2021.

[32] NHS England . About Digital Readiness Education. NHS Engl. Workforce Train. Educ. Digit. Transform, 2023. Available: https://digital-transformation.hee.nhs.uk/about-digital-readiness-education/about-digital-readiness-education [Accessed 1 Jul 2024].

[33] NHS Transformation Directorate . Mental Health Digital Playbook. NHS Transform Dir, 2021. Available: https://transform.england.nhs.uk/key-tools-and-info/digital-playbooks/mental-health-digital-playbook/ [accessed 1 Jul 2024].

[34] Blease C , Worthen A , Torous J . Psychiatrists’ experiences and opinions of generative artificial intelligence in mental healthcare: An online mixed methods survey. Psychiatry Res 2024;333:115724. 10.1016/j.psychres.2024.115724 38244285

[35] De Witte NAJ , Carlbring P , Etzelmueller A , et al . Online consultations in mental healthcare during the COVID-19 outbreak: An international survey study on professionals’ motivations and perceived barriers. Internet Interv 2021;25:100405. 10.1016/j.invent.2021.100405 34401365 PMC8350604

[36] Smith KA , Hardy A , Vinnikova A , et al . Digital Mental Health for Schizophrenia and Other Severe Mental Illnesses: An International Consensus on Current Challenges and Potential Solutions. JMIR Ment Health 2024;11:e57155. 10.2196/57155 38717799 PMC11112473

[37] Titov N , Hadjistavropoulos HD , Nielssen O , et al . From Research to Practice: Ten Lessons in Delivering Digital Mental Health Services. J Clin Med 2019;8:1239. 10.3390/jcm8081239 31426460 PMC6722769

[38] Kwon S , Firth J , Joshi D , et al . Accessibility and availability of smartphone apps for schizophrenia. Schizophrenia (Heidelb) 2022;8:98. 10.1038/s41537-022-00313-0 36385116 PMC9668219

[39] Shei R-J , Holder IG , Oumsang AS , et al . Wearable activity trackers-advanced technology or advanced marketing? Eur J Appl Physiol 2022;122:1975–90. 10.1007/s00421-022-04951-1 35445837 PMC9022022

[40] Sawyer C , Hassan L , Sainsbury J , et al . Using digital technology to promote physical health in mental healthcare: A sequential mixed-methods study of clinicians’ views. Early Interv Psychiatry 2024;18:140–52. 10.1111/eip.13441 37318221

[41] Rodriguez-Villa E , Rozatkar AR , Kumar M , et al . Cross cultural and global uses of a digital mental health app: results of focus groups with clinicians, patients and family members in India and the United States. Glob Ment Health 2021;8:e30. 10.1017/gmh.2021.28 PMC8392688 34512999

[42] Zweifel P . Innovation in health care through information technology (IT): The role of incentives. Soc Sci Med 2021;289:114441. 10.1016/j.socscimed.2021.114441 34592541

