# Supplementary material for: Healthcare professionals’ views on implementing digital health tools in psychosis: a national survey in the UK
Source: BMJ Digit Health Ai. 2025 Jun 11;1(1):e000005. doi: 10.1136/bmjdhai-2025-000005 (PMC13528399; doi:10.1136/bmjdhai-2025-000005)
Supplement: Supplementary file 1 [file bmjdhai-1-1-bmjdhai-2025-000005-s001.pdf]

Supplementary materials

Table of Contents

Supplementary Table 1. Healthcare Professional Survey 2

Supplementary Table 2. Tools staff would like to see implemented (total number of references = 114) 11

Supplementary Table 3. Reasons for supporting using digital health tools (total number of references = 285) 12

Supplementary Table 4. Reasons for not supporting using digital health tools (DHTs) (total number of references = 80) 13

Supplementary Table 5. Reasons for service user not benefiting from using Digital Health Technolgies (DHTs) (total number of references = 361) 14

Supplementary Table 6. Perceived potential advantages of apps and wearables in clinical practice: N (%) 16

Supplementary Table 7. Staff's opinions on using remote symptom monitoring and passive sensing in clinical practice: N (%) 17

Supplementary Table 8. Concerns and comments on using active symptom monitoring in clinical practice (total number of references = 213) 18

Supplementary Table 9. Concerns and comments about using passive sensing in clinical practice (total number of references = 173) 20

Supplementary Table 1. Healthcare Professional Survey

Demographics

**1. *Please select your gender***<br>Do you identify as:<br>❑ Man / Male<br>❑ Woman / Female<br>❑ Non-binary/third gender<br>❑ Prefer not to say/unsure<br>❑ Other (please state): …………………………<br>***Does your gender match with your sex assigned at birth?***<br>❑ Yes<br>❑ No<br>❑ Prefer not to say	***2. How old are you?***<br>………………………………………………………
**3. *What is your ethnic origin?***<br>**White**<br>❑ English, Welsh, Scottish, Northern Irish or British<br>❑ Irish<br>❑ Gypsy or Irish Traveller<br>❑ Roma<br>❑ Any other White background<br>**Asian or Asian British**<br>❑ Indian<br>❑ Pakistani<br>❑ Bangladeshi<br>❑ Chinese<br>❑ Any other Asian background<br>**Black, Black British, Caribbean or African**<br>❑ Caribbean<br>❑ African<br>❑ Any other Black, Black British, or Caribbean background<br>**Mixed or multiple ethnic groups**<br>❑ White and Black Caribbean<br>❑ White and Black African<br>❑ White and Asian<br>❑ Any other Mixed or multiple ethnic background	***4. What is your highest completed level of education?***<br>❑ Primary school<br>❑ Secondary school<br>❑ Diploma or equivalent<br>❑ Trade/technical/vocational training<br>❑ University bachelor’s degree<br>❑ University master’s degree<br>❑ PhD or higher<br>❑ Prefer not to say

**Other ethnic group**<br><br>❑ Arab<br>❑ Any other ethnic group<br>❑ Prefer not to say	
5. What is your job title?<br>❑ Mental health nurse<br>❑ Social worker<br>❑ Support worker / nursing assistant / STR worker / healthcare assistant<br>❑ Psychotherapist (or trainee)<br>❑ Psychiatrist (consultant, trainee, non-training grade)<br>❑ Psychologist (qualified, assistant, trainee)<br>❑ Allied health professional (please state) ………………………….<br>❑ Student nurse / student social worker / medical student / student allied health professional<br>❑ Prefer not to say<br>❑ Other (please state) …………………………………………….	***6. How long have you worked in your current role (in years)?***<br><br>…...

Technology Use Questions

<u>What are Digital Health Tools?</u>

In this survey we are asking about apps, sensing features on a smartphone, wearable devices or smartwatches that are used for things like monitoring symptoms, counting steps, helping with sleep routine, and setting medication reminders. We are interested in how these devices are used for things like sleep, mental health tracking, heart rhythm or location (whereabouts).

7. What percentage of service users on your caseload do you think would benefit from digital health tools (e.g. smartphone apps, wearable devices/smartwatches) to support their mental health (including those who already use them)?

☐	☐	☐	☐	☐
0%	25%	50%	75%	100%

a. If 0% to 75%...
Please tell us why you think some service users you work with would not benefit from digital health tools
……
……

Implementing Digital Health Tools into Clinical Practice

8. What training or infrastructure would you need to use digital health tools (e.g. smartphone apps, wearable devices/smartwatches) in your clinical practice? (Select all that apply):

- ❑ Regular training (updates)
- ❑ One off training session
- ❑ Funding
- ❑ Support from the clinical team
- ❑ Support from management
- ❑ Ongoing technical support (regarding using the device)
- ❑ Someone specifically employed to support staff and service users in how to use digital devices and interpret data flows (A 'Digital Navigator')

a. Is there any other training or infrastructure you would need/want?

……………………………………………..…………………………………………………………………… …………...

9. Would you like to see digital health tools (e.g. smartphone apps, wearable devices/smartwatches) implemented/used in your service?

- ❑ Yes
- ❑ No
- ❑ Not Sure
- ❑ Other …… ….

a. If yes…
Why? …… …………….
What tools would you like to see implemented? ……………………………………………………….

b. If No/Not sure
Why? …… …………….

10. What functions would you like to see digital health tools used for in clinical practice (Please tick to indicate)?

**Question**	**What digital health tool would you like to be used here?**			
	**Smartphone App**	**Wearable Device**	**Neither**	**Other. Please state**

I would like a digital health tool to alert/remind service users about healthcare appointments	☐	☐	☐	....
I would like a digital health tool to alert/remind service users to take their medication	☐	☐	☐	....
I would like a digital health tool to prompt service users to record how they are feeling	☐	☐	☐	....
I would like a digital health tool to remind service users to complete homework (between session)/at home activities	☐	☐	☐	....

The next few questions are about smartphone apps.

11. To what extent do you agree or disagree (1 = strongly disagree; 5 = strongly agree) that the following are potential advantages of smartphone apps for mental healthcare? (Please select all that apply)

**Question**	**Strongly disagree**	**Disagree**	**Neutral**	**Agree**	**Strongly agree**
Health-related apps can help service users take control over their mental health	1	2	3	4	5
Apps allow for more honest and valid data to be collected from a service user compared to administering questionnaires or asking service users directly about their symptoms	1	2	3	4	5
Apps provide service users the opportunity to record and reflect on symptoms and experiences over time	1	2	3	4	5
Apps provide the opportunity for service users to share information in 'real-time'	1	2	3	4	5

with their clinical team					
Apps provide the opportunity for service users to identify triggers and patterns	1	2	3	4	5
Service users may feel that information in an app is more private compared to talking to a member of their clinical team	1	2	3	4	5
Apps might help reduce health inequalities by enabling access to services in new ways	1	2	3	4	5

12. Please list any other reasons why, in your opinion, service users might want to use an app to manage their mental health.

……
……
………………………………………………

13. Do you have any ideas for any other ways that an app could help service users manage their mental health?

……
……
………………………………………………

14. Do you have any ideas for ways in which apps can help support your clinical practice / ways of working with service users?

……
……
………………………………………………

The next few questions are about wearable devices (e.g. Apple Watch or Fitbit)

15. To what extent do you agree or disagree (1 = strongly disagree; 5 = strongly agree) that the following are potential advantages of wearable devices in mental healthcare? (Please select all that apply)

**Question**	**Strongly disagree**	**Disagree**	**Neutral**	**Agree**	**Strongly agree**
Wearable devices can help service users take control over their mental health	1	2	3	4	5
Wearable devices allow for more honest and valid data to be collected from a service user compared to administering measures or asking service users directly about their symptoms	1	2	3	4	5
Wearable devices provide service users the opportunity to record and reflect on symptoms and experiences over time	1	2	3	4	5
Wearable devices provide service users the opportunity to share information in 'real-time' with their clinical team	1	2	3	4	5
Wearable devices provide service users the opportunity to identify triggers and patterns	1	2	3	4	5
Service users may feel that information collected by a wearable device is more private compared to talking to a member of their clinical team	1	2	3	4	5
Wearable devices might help reduce health inequalities by enabling access to services in new ways	1	2	3	4	5

16. Please list any other reasons why, in your opinion, service users might want to use a wearable device to manage their mental health.

………
………
………………………………………………

17. Do you have any ideas for any other ways that a wearable device could help service users manage their mental health?

Zhang X, *et al. BMJ Digit Health* 2025; 1:e000005. doi: 10.1136/bmjdhai-2025-000005

……
……
………………………………………………

18. Do you have any ideas for ways in which wearable devices can help support your clinical practice / ways of working with service users?

……
……
………………………………………………

How Digital Health Tools Collect and Use Data

19. Remote Symptom Monitoring

One-way smartphones can be used is to keep track of service users' symptoms or how they have been feeling day-to-day. This is called 'remote monitoring'.

How this works is the app prompts the user each day to answer questions about mood and experiences (like whether they've been feeling low, and whether they've been bothered by voices). It can also ask users about where they are and who they're with, to check out whether certain situations, places or people change are influencing how they are feeling.

The app can send the answers to these questions electronically to a service users' mental health team. That way, the team can explore if the service user needs any extra support, like an extra visit or an appointment with a member of the clinical team.

We are now going to ask your views on the use of remote symptom monitoring in clinical practice (i.e., direct work with service users).

**Question**	**Strongly disagree**	**Disagree**	**Neither agree nor disagree**	**Agree**	**Strongly agree**
Remote symptom monitoring would be time-consuming in clinical practice	1	2	3	4	5
Remote symptom monitoring would be too costly in clinical practice	1	2	3	4	5
Remote symptom monitoring would take the focus away from other needs of the service (money or time better spent elsewhere)	1	2	3	4	5
Remote symptom monitoring in clinical practice would be a burden on staff	1	2	3	4	5

Remote symptom monitoring in clinical practice would be concerning/worrisome because of data privacy or storage of personal data	1	2	3	4	5
Remote symptom monitoring in clinical practice would be difficult for staff who are not familiar with digital health tools	1	2	3	4	5
Remote symptom monitoring in clinical practice would be difficult for service users who are not familiar with digital health tools	1	2	3	4	5

a. How comfortable do you feel about using a remote monitoring app that collects information on service user's feelings or general health in your clinical practice?

☐ Comfortable ☐ Uncomfortable Uns☐

b. How comfortable do you feel about using a remote monitoring app that collects information on service user's behaviours or whereabouts in your clinical practice?

☐ Comfortable ☐ Uncomfortable Uns☐

c. Do you have any comments or concerns about using remote monitoring in your clinical work?

……..

20. Passive Sensing

Smartphones and wearables (like Fitbits or smartwatches) can gather lots of round-the-clock information, without a person having to *do* anything apart from wear the wearable or carry their phone around with them. This is called passive sensing.

This information tells a story about the kind of things a person has been doing – like whether they've been sleeping, exercising, travelling, or phoning people – and can give clues about how they might be feeling. For example, someone who is feeling very anxious or low might not feel like going out much or seeing people. They might stay at home more than usual and phone their friends less.

Because their smartphone can gather information about where they've been and how many phone calls they've made, it could quickly spot that change. Allowing their phone to spot changes like this could help the person (and their mental health team) know that they might need some extra support.

We are now going to ask your views on the use of passive sensing in clinical practice.

Question	Strongly disagree	Disagree	Neither agree nor disagree	Agree	Strongly agree

Reviewing and using passive sensing data would be time-consuming in clinical practice	1	2	3	4	5
Implementing passive sensing would be too costly in clinical practice	1	2	3	4	5
Collecting information using passive sensing would take the focus away from other needs of the service (money or time better spent elsewhere)	1	2	3	4	5
Passive sensing in clinical practice would be a burden on staff	1	2	3	5	5
Passive sensing in clinical practice would be concerning/worrisome because of data privacy or storage of personal data	1	2	3	4	5
Passive sensing in clinical practice would be difficult to use/make sense of for staff who are not familiar with digital health tools	1	2	3	4	5
Passive sensing in clinical practice would be difficult for service users who are not familiar with digital health tools	1	2	3	4	6

a. How comfortable do you feel about *passively* collecting information (through a wearable device or a mobile phone) about service user's feelings or general health in your clinical practice?

☐ Comfortable ☐ Uncomfortable Uns☐

b. How comfortable do you feel about *passively* collecting information (through a wearable device or a mobile phone) about service user's behaviours or whereabouts in your clinical practice?

☐ Comfortable ☐ Uncomfortable Uns☐

c. Do you have any comments or concerns about passively collecting information in your clinical practice?

………
……………….

Supplementary Table 2. Tools staff would like to see implemented (total number of references = 114)

Coding	Number of coding references, n (%)
App	41 (35.96)
Wearable devices	32 (28.07)
Tablets	24 (21.05)
Smartphones	13 (11.40)
Virtual Reality	2 (1.75)
Online tools	1 (0.88)
Videoconference	1 (0.88)

Zhang X, *et al. BMJ Digit Health* 2025; 1:e000005. doi: 10.1136/bmjdhai-2025-000005

Supplementary Table 3. Reasons for supporting using digital health tools (total number of references = 285)

Coding	Number of coding references, n (%)
Benefits service users	57 (20.00)
Supports service delivery, cope with staff shortage	34 (11.93)
Provides more information and objective data	31 (10.88)
Promotes self-management	26 (9.12)
Improves engagement with care	20 (7.03)
Easy access	19 (6.67)
Service users in control of their mental health and life	19 (6.67)
Keep track of mental health	13 (4.56)
People use technology in daily life	13 (4.56)
Simple to use	13 (4.56)
New development, innovation	10 (3.51)
More options for mental healthcare	9 (3.16)
Useful for physical health	6 (2.11)
Saves time	5 (1.75)
More connected	3 (1.05)
Already use technology in services	2 (0.70)
Saves costs	2 (0.70)
Technology is widely used in other services	2 (0.70)
Reduces stigma	1 (0.35)

Supplementary Table 4. Reasons for not s We do, however, acknowledge that the use cases provided for this item were limited and not exhaustive, which may have influenced participants' responses. A broader selection of potential applications might have yielded different preferences, reflecting a wider range of clinician priorities. **upporting using digital health tools (DHTs) (total number of references = 80)**

Coding	Number of coding references, n (%)
Need more information and evidence	11 (13.75)
Not cost-effective	10 (12.50)
Not useful	9 (11.25)
Concerns about its feasibility	7 (8.75)
Don't want to replace face-to-face care	6 (7.50)
Not a good fit for specific settings	6 (7.50)
Inability to use DHTs (both service user and staff)	5 (6.25)
Increases workload	5 (6.25)
Service user won't engage or be interested	5 (6.25)
Concerns about privacy and safety	4 (5.00)
Might exacerbate symptoms	3 (3.75)
Impersonal	3 (3.75)
Already plenty of apps out there	2 (2.50)
DHTs not a solution for staff shortage	2 (2.50)
Don’t think it’s the priority	2 (2.50)

Zhang X, *et al. BMJ Digit Health* 2025; 1:e000005. doi: 10.1136/bmjdhai-2025-000005

Supplementary Table 5. Reasons for service user not benefiting from using Digital Health Technolgies (DHTs) (total number of references = 361)

Coding	Number of coding references
Inability to use DHTs	78 (21.61)
Paranoia associated with technology	46 (12.74)
Poor engagement with DHTs	32 (8.86)
Distressed by using technology	28 (7.76)
Level of illness	21 (5.82)
Age	18 (4.99)
Not interested in using technology	15 (4.16)
Cost	14 (3.88)
Lack of access to digital technology	13 (3.60)
Anxious about using digital devices	11 (3.05)
Prefer face-to-face services	11 (3.05)
Lack of motivation	9 (2.49)
Lack of therapeutic relationship	7 (1.94)
Likely to lose, damage, or sell the device	5 (1.39)
Not seeing the benefit	5 (1.39)
Technology overuse	5 (1.39)
Chaotic lifestyles	4 (1.11)
Mistrust of technology	4 (1.11)
Data protection concerns	3 (0.83)
Ineffectiveness	3 (0.83)
Inpatient setting	3 (0.83)
Lack of awareness of DHTs	3 (0.83)
No intervention will work for everyone	3 (0.83)
Obsess about monitoring results	3 (0.83)
Lack of insight	2 (0.55)
Not relevant to treatment	2 (0.55)
Not reliable	2 (0.55)
Not personalise	2 (0.55)
Availability of DHTs	1 (0.28)
Lack of continuity of digital health services	1 (0.28)
Don't want to be monitored	1 (0.28)
Inequity	1 (0.28)

Intrusive	1 (0.28)
Not trust service	1 (0.28)
Risk of online safety	1 (0.28)
Risk of self-harm	1 (0.28)
Staff lack of knowledge of DHTs	1 (0.28)

Zhang X, *et al. BMJ Digit Health* 2025; 1:e000005. doi: 10.1136/bmjdhai-2025-000005

Supplementary Table 6. Perceived potential advantages of apps and wearables in clinical practice: N (%)

Items	Apps						Wearable devices					
	**Strongly disagree**	**Disagree**	**Neutral**	**Agree**	**Strongly agree**	**NA**	**Strongly disagree**	**Disagree**	**Neutral**	**Agree**	**Strongly agree**	**NA**
They can help service users take control over their mental health	9 (2.56)	15 (4.26)	69 (19.6)	204 (57.95)	53 (15.06)	2 (0.57)	11 (3.12)	21 (5.97)	119 (33.81)	170 (48.3)	29 (8.24)	2 (0.57)
They allow for more honest and valid data to be collected from a service user compared to administering questionnaires or asking service users directly about their symptoms	9 (2.56)	40 (11.36)	96 (27.27)	158 (44.89)	47 (13.35)	2 (0.57)	10 (2.84)	30 (8.52)	118 (33.52)	161 (45.74)	31 (8.81)	2 (0.57)
They provide service users the opportunity to record and reflect on symptoms and experiences over time	5 (1.42)	9 (2.56)	32 (9.09)	244 (69.32)	60 (17.05)	2 (0.57)	11 (3.12)	16 (4.55)	89 (25.28)	203 (57.67)	31 (8.81)	2 (0.57)
They provide the opportunity for service users to share information in 'real-time' with their clinical team	8 (2.27)	16 (4.55)	51 (14.49)	212 (60.23)	63 (17.9)	2 (0.57)	11 (3.12)	23 (6.53)	90 (25.57)	190 (53.98)	35 (9.94)	3 (0.85)
They provide the opportunity for service users to identify triggers and patterns	6 (1.7)	4 (1.14)	37 (10.51)	239 (67.9)	64 (18.18)	2 (0.57)	9 (2.56)	16 (4.55)	78 (22.16)	216 (61.36)	31 (8.81)	2 (0.57)
Service users may feel that information in it is more private compared to talking to a member of their clinical team	18 (5.11)	56 (15.91)	129 (36.65)	119 (33.81)	28 (7.95)	2 (0.57)	18 (5.11)	68 (19.32)	130 (36.93)	114 (32.39)	20 (5.68)	2 (0.57)
They might help reduce health inequalities by enabling access to services in new ways	16 (4.55)	37 (10.51)	102 (28.98)	156 (44.32)	38 (10.8)	3 (0.85)	21 (5.97)	47 (13.35)	118 (33.52)	139 (39.49)	24 (6.82)	3 (0.85)

Note: The cells are colour-coded, with darker blue indicating a higher percentage.

Supplementary Table 7. Staff's opinions on using remote symptom monitoring and passive sensing in clinical practice: N (%)

Items	Remote symptom monitoring						Passive sensing					
	Strongly disagree	Disagree	Neutral	Agree	Strongly agree	NA	Strongly disagree	Disagree	Neutral	Agree	Strongly agree	NA
Reviewing and using data would be time-consuming	17 (4.83)	83 (23.58)	114 (32.39)	115 (32.67)	21 (5.97)	2 (0.57)	17 (4.83)	53 (15.06)	119 (33.81)	126 (35.8)	33 (9.38)	4 (1.14)
Implementing it would be too costly	14 (3.98)	65 (18.47)	161 (45.74)	91 (25.85)	19 (5.4)	2 (0.57)	14 (3.98)	44 (12.5)	173 (49.15)	92 (26.14)	25 (7.1)	4 (1.14)
Collecting information using it would take the focus away from other needs of the service (money or time better spent elsewhere)	17 (4.83)	97 (27.56)	141 (40.06)	81 (23.01)	14 (3.98)	2 (0.57)	15 (4.26)	80 (22.73)	150 (42.61)	79 (22.44)	24 (6.82)	4 (1.14)
It would be a burden on staff	23 (6.53)	97 (27.56)	116 (32.95)	95 (26.99)	19 (5.4)	2 (0.57)	14 (3.98)	88 (25)	135 (38.35)	87 (24.72)	23 (6.53)	5 (1.42)
It would be concerning/worrisome because of data privacy or storage of personal data	15 (4.26)	79 (22.44)	129 (36.65)	105 (29.83)	22 (6.25)	2 (0.57)	12 (3.41)	68 (19.32)	121 (34.38)	107 (30.4)	40 (11.36)	4 (1.14)
It would be difficult to use/make sense of for staff who are not familiar with digital health tools	10 (2.84)	35 (9.94)	91 (25.85)	177 (50.28)	37 (10.51)	2 (0.57)	7 (1.99)	37 (10.51)	85 (24.15)	180 (51.14)	39 (11.08)	4 (1.14)
It would be difficult for service users who are not familiar with digital health tools	7 (1.99)	19 (5.4)	66 (18.75)	200 (56.82)	58 (16.48)	2 (0.57)	8 (2.27)	41 (11.65)	86 (24.43)	163 (46.31)	49 (13.92)	5 (1.42)

Note: The cells are colour-coded, with darker blue indicating a higher percentage.

Zhang X, *et al. BMJ Digit Health* 2025; 1:e000005. doi: 10.1136/bmjdhai-2025-000005

Supplementary Table 8. Concerns and comments on using active symptom monitoring in clinical practice (total number of references = 213)

Coding	Number of coding references, n (%)
Service users feel intrusive and controlling	23 (10.80)
Service user paranoid or anxious about technology	20 (9.39)
Staff don't have capacity to respond	18 (8.45)
Data protection concerns	17 (7.98)
Staff feel uncomfortable with location tracking	16 (7.51)
Need consent of the individual	8 (3.76)
Service users may be concerned about privacy	8 (3.76)
Reduces human contact	8 (3.76)
Need training (both staff and service users)	7 (3.29)
Service users may underreport or overreport symptoms	7 (3.29)
Staff feel comfortable with using it as an addition to standard care	7 (3.29)
Staff need more research, information, evidence to support decision	7 (3.29)
Clear guidelines and expectations for service users and clinicians	6 (2.82)
Unsuitable for some population or clinical settings	6 (2.82)
Active symptom monitoring is useful for mental healthcare	6 (2.82)
Service users may overly reliant on technology for care	5 (2.35)
Staff feel uncomfortable with tracking service users	4 (1.88)
Staff unclear who is responsible for the results of monitoring	4 (1.88)
Self-monitoring reliant on service user engagement to be useful	4 (1.88)
Useful to identify triggers and patterns	4 (1.88)
Increase staff workload	3 (1.41)
Service users may misuse the technology	3 (1.41)
Negative impacts on therapeutic relationship	3 (1.41)
Data should be easy for the clinical team to understand	2 (0.94)
Staff concerned about ethical issues	2 (0.94)
Active monitoring may collect inaccurate information	2 (0.94)
The volume of data would be unmanageable for staff	2 (0.94)
Real time recording, better than relying on memory	2 (0.94)
Staff may struggle with technology	1 (0.47)
Adoption curve for services	1 (0.47)
Not cost-effective	1 (0.47)
Service users feel connected with care team	1 (0.47)
Good for physical conditions, not for mental health conditions	1 (0.47)

Risks of users to loss, damage, misuse of device	1 (0.47)
Monitoring may cause distress to service user	1 (0.47)
Technology is harmful in itself	1 (0.47)

Supplementary Table 9. Concerns and comments about using passive sensing in clinical practice (total number of references = 173)

Coding	Number of coding references, n (%)
Intrusive and controlling	26 (15.03)
Issue of consent	25 (14.45)
Could increase anxiety and paranoia	16 (9.25)
Data protection concerns	9 (5.20)
Feels uncomfortable to track service users in this way	9 (5.20)
Uncomfortable with collecting location data	9 (5.20)
Inaccurate and lack of context	8 (4.62)
Need training to use	8 (4.62)
Increase workload	8 (4.62)
Service user should control the data and decide who to share it with	5 (2.89)
As an add-on to face-to-face service	4 (2.31)
Not necessary and raises ethical questions	4 (2.31)
Too much data	4 (2.31)
Who would be responsible for checking, responding to the data?	4 (2.31)
Useful for identifying patterns and triggers	4 (2.31)
Data collected needs to be specific, accountable and justifiable	3 (1.73)
Face-to-face is more appropriate	2 (1.16)
A good idea	2 (1.16)
Overly rely on information from the app	2 (1.16)
Service users do not need to be monitored to this level	2 (1.16)
Service users don't want to be monitored	2 (1.16)
Unsure if necessary in particular settings	2 (1.16)
Service user’s capacity and understanding of passive collecting of information	1 (0.58)
May damage therapeutic relationship	1 (0.58)
Real-time feedback	1 (0.58)
Empower and support self-management	1 (0.58)
Good for sleep monitoring	1 (0.58)
Good for service user who does not want to talk	1 (0.58)
Homework and appointment reminder	1 (0.58)
Needs to be careful about the interpretation of the data	1 (0.58)

More objective evidence	1 (0.58)
Not useful	1 (0.58)
Passive sensing more practical	1 (0.58)
Personalised implementation	1 (0.58)
Should be used collaboratively	1 (0.58)
Shouldn't be a burden	1 (0.58)
Stigma	1 (0.58)
